# Bidirectional association between functional disability and multimorbidity among middle-aged and older adults in Thailand

**DOI:** 10.3389/fpubh.2022.1055699

**Published:** 2022-12-05

**Authors:** Supa Pengpid, Karl Peltzer, Dararatt Anantanasuwong

**Affiliations:** ^1^Department of Health Education and Behavioral Sciences, Faculty of Public Health, Mahidol University, Bangkok, Thailand; ^2^Department of Public Health, Sefako Makgatho Health Sciences University, Pretoria, South Africa; ^3^Department of Healthcare Administration, College of Medical and Health Science, Asia University, Taichung, Taiwan; ^4^Department of Psychology, University of the Free State, Bloemfontein, South Africa; ^5^Department of Psychology, College of Medical and Health Science, Asia University, Taichung, Taiwan; ^6^Center for Aging Society Research (CASR) at National Institute of Development Administration (NIDA), Bangkok, Thailand

**Keywords:** multimorbidity, functional disability, longitudinal study, Thailand, bidirectional

## Abstract

**Objectives:**

The purpose of this study was to assess the bidirectional association between multimorbidity (MM) and functional disability among middle-aged and older adults in a longitudinal study in Thailand.

**Methods:**

We analyzed longitudinal data of participants aged 45 years and older from two consecutive waves (in 2015 and 2017) of the Health, Aging, and Retirement in Thailand (HART). Functional disability was assessed with a 4-item activity of daily living (ADL) scale. Logistic regression analysis was conducted to assess the association between baseline functional disability and incident MM (≥2), and baseline morbidity and incident functional disability.

**Results:**

The results indicate that a total of 1,716 individuals without morbidity at baseline and 3,529 without functional disability at baseline were included. At follow-up, 16.7 and 20.0% of functional disability cases and 7.1 and 3.6% of nonfunctional disability cases developed 2 morbidities and 3 or more morbidities, respectively, and 6.6% of MM cases and 4.0% of non-MM cases developed a functional disability. In the final logistic regression model adjusted for education, income, age, marital status, sex, smoking tobacco, body mass index (BMI), alcohol use, physical activity, and social engagement, functional disability at baseline was positively associated with incident MM (≥2) (adjusted odds ratio [aOR]: 2.58, 95% CI: 1.42–4.72), and MM (≥3) at baseline was positively associated with incident functional disability (aOR: 1.97, 95% CI: 1.13–3.43).

**Conclusion:**

Multimorbidity and functional disability were bidirectionally associated.

## Introduction

There has been a demographic and epidemiological transition that has increased aging and chronic noncommunicable diseases in low- and middle-income countries (LMICs) (1) such as Thailand (2–5). This may include multimorbidity (MM) (co-existence of ≥2 chronic conditions) and functional disability in LMICs, including Thailand, which are an increasing burden on the healthcare systems (6–13). In a study among aging adults in six LMICs (i.e., China, India, Ghana, South Africa, Mexico, and Russia), the prevalence of MM was 45.5% (based on conditions: “angina, arthritis, asthma, chronic back pain, chronic lung disease, diabetes, edentulism, hearing problems, hypertension, stroke, visual impairment”) (14), and among older adults (≥50 years) in the six LMICs, the prevalence of MM (based on “arthritis, stroke, angina, diabetes, chronic lung disease, asthma, and hypertension”) was 17.4% in China, 25.2% in India, 16.6% in Ghana, 23.4% in South Africa, 45.3% in Mexico, and 23.6% in Russia (15). In a small community-based study among older adults (≥60 years) in southern Thailand, the prevalence of MM was 16.8% (16) and in national surveys, among older adults (≥60 years) in 2007 the prevalence of MM was 14.7% (“hypertension, heart disease, diabetes, cancer, stroke, and paralysis”) (17) and in 2018, 30.4% (MM: “cancer, diabetes, hypertension, stroke, asthma, or another self-named chronic disease) (18).

According to the World Health Organization (19), 15% of the global population is estimated to be disabled in 2011 (19). Among older adults (≥50 years) in the six LMICs, the prevalence of functional disability (1+ activities of daily living [ADLs] limitation) ranged from 16.2% in China to 55.7% in India (15). In a national survey among people aged 60 years and older in Thailand in 1997, the prevalence of long-term disability was 19% (20). Among older adults (60 years) in Thailand in 2011, 2.7% had difficulty with the ADL item dressing and 2.2% with eating (21); in 2014 and 2017, 7.6% had difficulty with at least one (of 8) ADL item (22, 23). In a small study in rural Thailand, the prevalence of severe disability among older adults was 11.9% (24).

Multimorbidity is implicated in various negative health outcomes, such as increased mental morbidity, disability, increased healthcare utilization, adverse drug events, and death (7, 25–27). Similarly, functional disability is associated with increased hospitalization (28), poor self-rated quality of life (29), and mortality (30, 31). Previous studies have shown that demographic factors, such as older age and female sex (32), lower socioeconomic status (6, 9), and lifestyle factors, such as smoking (33), alcohol use (32, 33), physical inactivity (32, 34), body weight status and obesity (32, 35), and lack of social engagement (36, 37) were associated with MM. Furthermore, demographic factors, such as older age (15, 29) and female sex (29, 38, 39), lower socioeconomic status (38, 40), lifestyle factors, such as smoking (41), alcohol use (42), physical inactivity (40, 43, 44), body weight status and obesity (40, 41), low social capital (15) and low social interaction (44) were associated with functional disability.

Most studies have investigated MM and incident functional disability (45–48), but few studies have investigated functional disability and incident MM, and bidirectional associations between MM and functional disability (7, 49). A study on the bidirectional association between MM and functional disability among older adults in China (including the chronic conditions: “hypertension, diabetes, cancer, chronic lung disease, cardiovascular disease, emotional or psychiatric disease, stomach or other digestive diseases, arthritis or rheumatism, kidney disease, liver disease, memory-related disease, and asthma”) and Europe (including 9 diseases: “hypertension, diabetes, cancer, chronic lung disease, cardiovascular disease, emotional of psychiatric disease, stomach or duodenal ulcer, and arthritis or rheumatism”) (7) found that across 2 longitudinal studies, functional disability and MM were bidirectionally associated. In the China (CHARLS) and Europe (SHARE) study, nationally representative cohorts were followed from 2011 to 2015, showing that participants with ADL/IADL disability at baseline were at a higher risk of developing MM and people with MM at baseline were at a higher risk of developing ADL/IADL disability in a dose-response fashion (7). Several longitudinal studies have investigated the determinants of MM and functional disability separately (49, 50), rather than studying both simultaneously (49). It is suggested that shared modifiable risk for both MM and functional disability exist, which should be further investigated (49).

To gain better knowledge of the association between MM and functional disability in Southeast Asia, the aim of this study was to assess the bidirectional association between MM and functional disability among middle-aged and older adults in a longitudinal study in Thailand. In particular, the study had two objectives, namely, (1) to estimate the association between functional disability at baseline and incident MM and (2) to estimate the association between morbidity counts at baseline and incident functional disability.

## Methods

### Participants and procedure

This study analyzed longitudinal data from two consecutive waves (2015 and 2017) of the Health, Aging, and Retirement in Thailand (HART). In a national sample from five regions and Bangkok and its vicinity, one adult (≥45 years) per household was randomly selected using a multistage sampling design [refer to (51) for further details]. The 2015 (*N* = 5,616) and 2017 surveys included 3,708 members of the 2015 HART cohort (92 died during a follow-up or 4.3% of the baseline respondents were in the study area; 1,554 moved away from the study area; 270 declined participation; and the response rate: 72.3% and the retention rate: 66.03%). A total of 3,708 participants who responded to the 2015 and 2017 surveys were included in the study, and 3,646 had complete information on our variables of interest (MM and functional disability). Participants were interviewed at their homes by trained field workers using the paper-and-pencil (PAPI) questionnaire in wave 1 and computer-assisted personal interviewing (CAPI) in wave 2.

### Measures

#### Outcome variables

*Chronic physical conditions* were evaluated by self-reported healthcare provider diagnosed conditions, including hypertension, diabetes, lung diseases, emphysema, cardiovascular diseases, heart disease, heart failure, rheumatism, arthritis, bone diseases, low bone density, osteoporosis, kidney diseases, cancer, liver diseases, emotional/nervous or psychiatric disease, brain diseases, Alzheimer's disease, and visual and hearing impairment. MM was defined as having two or more chronic diseases, and non-MM as having none or one physical chronic disease.

*Functional disability* was measured based on a 4-item (eating, bathing, dressing, and washing) modified ADL index (52). Responses ranged from 0 = “able to do it all by myself” to 3 = “need help for all steps.” Functional disability was defined as any of the four items not being able to do all by themselves (Cronbach's α = 0.94 at wave 1 and 0.90 at wave 2).

#### Covariates

*Sociodemographic data* included educational level, sex, age, marital status, and income quartile. Education was grouped into (1) no formal education, (2) elementary school, and (3) more than elementary school (middle school, high school, vocational diploma or 2-year diploma degree, bachelor's degree, or higher than bachelor's degree). “The income quartile was calculated based on annual income from employment, own business, agricultural/livestock/fishing business, short-term or contract work, financial support from family, remuneration/pension income from the government fund, occupational pension fund, private pension fund, social security/welfare income, income e from government living allowance, veteran's welfare benefit, other welfare assistance income, and income from other sources, into four groups 1 = 0 to <13,000 Thai Baht, 2 = 13,000 to <50,000, 3 = 50,000 to <140,000, 4 = ≥140,000 Thai Baht (Average exchange rate in 2015: 1 US=34.2 Baht)” (53).

*Tobacco smoking* was sourced from the item, “Have you ever smoked cigarettes?” (response options: “1 = yes, and still smoke now, 2 = yes, but quit smoking, and 3 = never”).

*Alcohol use* was assessed with the question, “Have you ever drunk alcoholic beverages such as liquor, beer, or wine?” (response options: 1 = yes, and still drinking now, 2 = yes, but do not drink now, and 3 = never).

*Past week physical activity or exercise* (frequency: “How often do you exercise?” (days a week) and duration of any type: “On the day you exercise, how long do you exercise?” (minutes) (54)) was grouped into “none = inactivity, 1–149 min/week = low activity, and ≥150 min/week = high activity.” (55).

*Body mass index* (BMI) was based on self-reported body weight and height and was classified using Asian cutoff criteria into “underweight (<18.5 kg/m^2^), normal weight (18.5–22.9 kg/m^2^), overweight (23–24.9 kg/m^2^), and obesity (25+ kg/m^2^)” (56).

*Social engagement* included six items of formal and one item of informal social engagement (defined as at least one activity in the past month) (53, 57). Formal social engagement included religious, occupational, and cultural organizations; alumni or parent association or association of people from the same hometown; volunteer; and political organizations. Responses were coded as “1 = daily to at least once a month” and “0 = once a year or never.” Informal social engagement was determined with the following questions: (1) “In the past year, do you have any close friends or relatives who live nearby and have a close relationship with? (Please refer to the only person whom you meet most often)” and (2) “If so, how often do you meet with them in person (number of times per day, week, month, year, other, never)?” Informal social engagement was defined as “1 = having a close friend or relative who lives nearby and have a close relationship with and having met that person at least in the past 1 month” and “0 = not having a close friend or relative or meeting a close friend less than once a month in the past year” (53).

#### Data analysis

The proportion of older adults with incident MM (those who have MM at wave 2 and are without morbidity at wave 1) and incident functional disability (those who have a functional disability at wave 2 and are without functional disability at wave 1) is presented with frequencies and percentages. Pearson's chi-square tests are used to compare baseline characteristics among groups. The first logistic regression model estimated odds ratios (ORs) and confidence intervals (CIs) between functional disability at baseline and incident MM, and the second model compared morbidity counts at baseline and incident functional disability. Three models are presented for incident MM and incident functional disability. The first model is unadjusted; in the second model, adjustments are made for age, income, sex, education, and marital status, and in the third model, adjustments are made for model 2 variables plus smoking, physical activity, BMI, alcohol use, and social engagement. The selection of covariates is based on a previous review of the literature (6–8, 14, 15, 25, 29, 32–44). A value of *p* ≤ 0.05 was considered statistically significant. All statistical analyses were performed using StataSE version 15.0 (College Station, TX, USA).

## Results

### Baseline sample characteristics

The analytic baseline sample included 3,638 individuals aged 45 years and older. The prevalence of MM was 23.5%, and the prevalence of functional disability was 3.2%. Both MM and functional disability increased with age, decreased with higher education, decreased with higher income, decreased with alcohol use and smoking, and decreased with being married or cohabiting. MM was higher in women than in men but functional disability did not differ by sex. MM increased with increased body weight and functional disability was greater among those with underweight and who were physically inactive. The social engagement did not significantly differ by MM and functional disability (refer to Table 1).

**Table 1:** Baseline sample characteristics, HART, 2015.

**Variables**	**Subcategories**	**Sample**		**Morbidity**			**Functional disability**
			**1**	**2**	**≥3**	***P*-value**		***P*-value**
		**N (%)**	**N (%)**	**N (%)**	**N (%)**		**N (%)**	
All		3,638	1,098 (30.2)	596 (16.4)	260 (7.1)		115 (3.2)	
Age (in years)	45–54	672 (18.5)	149 (22.2)	58 (8.6)	14 (2.1)	<0.001	4 (0.6)	<0.001
							11 (1.1)	
							20 (2.2)	
	55–64	985 (27.1)	295 (29.9)	142 (14.4)	41 (4.2)		80 (7.6)	
	66–74	926 (25.5)	300 (32.4)	166 (17.9)	81 (8.7)			
	75 or more	1,055 (29.0)	354 (33.6)	230 (21.8)	124 (11.8)			
Sex	Female	1,942 (53.4)	610 (31.4)	358 (18.4)	156 (8.0)	< 0.001	62 (3.2)	0.907
	Male	1,696 (46.6)	488 (28.8)	238 (14.0)	104 (6.1)		53 (3.1)	
Education	None	238 (6.6)	69 (29.0)	66 (27.7)	24 (10.1)	<0.001	26 (10.9)	<0.001
	Elementary	2,811 (77.4)	877 (31.2)	464 (16.5)	213 (7.6)		77 (2.7)	
	>Elementary	582 (16.0)	149 (25.6)	65 (11.2)	23 (4.0)		12 (2.1)	
Marital status	Not married	1,478 (40.6)	471 (31.9)	274 (18.5)	132 (8.9)	<0.001	75 (5.1)	<0.001
	Married/cohabiting	2,159 (59.4)	627 (29.0)	322 (14.9)	128 (5.9)		40 (1.9)	
Income quartile	Low	867 (23.8)	275 (31.7)	180 (20.8)	81 (9.3)	<0.001	32 (3.7)	<0.001
	Lower middle	922 (25.3)	329 (35.7)	176 (19.1)	75 (8.1)		55 (6.0)	
	Upper middle	952 (26.2)	248 (26.1)	136 (14.3)	68 (7.1)		20 (2.1)	
	High	897 (24.7)	246 (27.4)	104 (11.6)	36 (4.0)		8 (0.9)	
Alcohol use	Never	2,939 (80.8)	917 (31.2)	507 (17.3)	213 (7.2)	<0.001	102 (3.5)	0.002
	Past	260 (7.1)	71 (27.3)	42 (16.2)	36 (13.8)		11 (4.2)	
	Current	439 (12.1)	110 (25.1)	47 (10.7)	11 (2.5)		2 (0.5)	
Smoking tobacco use	Never	2,912 (80.0)	915 (31.4)	498 (17.1)	210 (7.2)	<0.001	97 (3.3)	<0.001
	Past	287 (7.9)	91 (31.7)	47 (16.4)	33 (11.5)		16 (5.6)	
	Current	439 (12.1)	92 (21.0)	51 (11.6)	17 (3.9)		2 (0.2)	
Physical activity	None	2,112 (58.1)	599 (28.4)	362 (17.1)	158 (7.5)	0.180	94 (4.5)	<0.001
	1–149 min/week	935 (25.7)	309 (33.0)	144 (15.4)	63 (6.7)		18 (1.9)	
	≥150 min/week	591 (16.2)	190 (32.1)	90 (15.2)	39 (6.6)		3 (0.5)	
Body mass index	Normal	1,230 (37.5)	357 (29.0)	163 (13.3)	68 (5.5)	<0.001	39 (3.2)	<0.001
	Under	352 (10.7)	103 (29.3)	59 (16.8)	22 (6.3)		24 (6.8)	
	Overweight	653 (19.9)	206 (31.5)	99 (15.2)	41 (6.3)		11 (1.7)	
	Obesity	1,041 (31.8)	322 (30.9)	207 (19.9)	103 (9.9)		21 (2.0)	
Social engagement	No	242 (6.7)	69 (28.5)	50 (20.7)	21 (8.7)	0.177	9 (3.7)	0.609
	Yes	3394 (93.3)	1028 (30.3)	546 (16.1)	239 (7.0)		106 (3.1)	

### Incident sample characteristics

In the first model that estimates incident morbidity, a total of 1,716 individuals without morbidity were included from baseline, with 30 (1.8%) having a functional disability at baseline. At follow-up, 16.7% and 20.0% of functional disability cases and 7.1 and 3.6% of nonfunctional disability cases developed 2 morbidities and 3 or more morbidities, respectively. Middle-aged and older adults with functional disability at baseline had a significantly higher prevalence of morbidity counts at follow-up (*p* < 0.001). Those with higher incident morbidity counts were likely older, were unmarried, had less income, had no social engagement, and were not currently smoking tobacco or using alcohol than those without or with lower morbidity counts (refer to Table 2).

**Table 2:** Sample characteristics of participants with incident morbidity, Thailand, 2015–2017.

**Variables**	**Subcategories**	**Incident morbidity**				***P*-value**
		**0 (*n =* 1,118)**	**1 (*n =* 403)**	**2 (*n =* 123)**	**≥3 (*n =* 70)**	
		***N* (%)**	***N* (%)**	***N* (%)**	***N* (%)**	
Age (in years)	45–54	349 (76.5)	83 (18.2)	23 (5.0)	1 (0.2)	< 0.001
	55–64	357 (69.2)	118 (22.9)	29 (5.6)	12 (2.3)	
	66–74	231 (59.2)	99 (25.4)	35 (9.0)	25 (6.4)	
	75 or more	181 (51.4	103 (20.3)	36 (10.2)	32 (9.1)	
Sex	Female	560 (66.9)	179 (21.4)	60 (7.2)	38 (4.5)	0.196
	Male	558 (63.6)	224 (25.5)	63 (7.2)	32 (3.6)	
Education	None	51 (63.8)	20 (25.0)	3 (3.8)	6 (7.5)	0.409
	Elementary	839 (65.4)	293 (22.8)	97 (7.6)	54 (4.2)	
	>Elementary	226 (65.1)	88 (25.4)	23 (6.6)	10 (2.9)	
Marital status	Not married	377 (61.6)	148 (24.2)	50 (8.2)	37 (6.0)	0.006
	Married/cohabiting	740 (67.3)	254 (23.1)	73 (6.6)	33 (3.0)	
Income quartile	Low	229 (65.4)	74 (21.1)	29 (8.3)	18 (5.1)	0.036
	Lower middle	202 (58.9)	88 (25.7)	32 (9.3)	21 (6.1)	
	Upper middle	331 (65.5)	124 (24.6)	35 (6.9)	15 (3.0)	
	High	356 (69.0)	117 (22.7)	27 (5.2)	16 (3.1)	
Alcohol use	Never	858 (64.7)	307 (23.1)	101 (7.6)	61 (4.6)	0.044
	Past	67 (59.3)	34 (30.1)	6 (5.3)	6 (5.3)	
	Current	193 (70.4)	62 (22.6)	16 (5.8)	3 (1.1)	
Smoking tobacco use	Never	841 (64.1)	311 (23.7)	102 (7.8)	59 (4.5)	0.014
	Past	69 (59.0)	32 (27.4)	9 (7.7)	7 (6.0)	
	Current	208 (73.2)	60 (21.1)	12 (4.2)	4 (1.4)	
Physical activity	None	667 (65.7)	231 (22.8)	77 (7.6)	40 (3.9)	0.842
	1–149 min/week	270 (64.3)	100 (23.8)	30 (7.1)	20 (4.8)	
	≥150 min/week	181 (64.9)	72 (25.8)	16 (5.7)	10 (3.6)	
Body mass index	Normal	433 (66.7)	149 (23.0)	47 (7.2)	20 (3.1)	0.855
	Under	109 (63.7)	42 (24.6)	11 (6.4)	9 (5.3)	
	Overweight	197 (63.3)	79 (25.4)	20 (6.4)	15 (4.8)	
	Obesity	274 (65.9)	92 (22.1)	33 (7.9)	17 (4.1)	
Social engagement	No	58 (56.9)	24 (23.5)	16 (15.7)	4 (3.9)	0.007
	Yes	1,059 (65.9)	377 (23.4)	107 (6.7)	65 (4.0)	
Functional disability	No	1,092 (66.0)	386 (23.3)	117 (7.1)	59 (3.6)	<0.001
	Yes	10 (33.3)	9 (30.0)	5 (16.7)	6 (20.0)	

In the second model that estimates incident functional disability, a total of 3,529 individuals without a functional disability were included from baseline, with 1,115 (30.1%), 607 (16.4%), and 270 (7.3%) having 1, 2, and 3 or more morbidities at baseline. At follow-up, 6.6% of MM cases and 4.0% of non-MM cases developed a functional disability. Furthermore, 3.4% of 0, 4.9% of 1, 5.5% of 2, and 9.4% of 3 or more morbidity cases developed a functional disability. Middle-aged and older adults with physical MM at baseline had a significantly higher prevalence of functional disability at follow-up (*p* < 0.002), and those with higher morbidity counts at baseline had a significantly higher prevalence of functional disability at follow-up (*p* < 0.001). Those with incident functional disability were likely to be older, had lower education, were unmarried, had lower income, were not currently using alcohol, were less physically active, and were more likely underweight than those without functional disability (refer to Table 3).

**Table 3:** Sample characteristics of participants with incident functional disability, Thailand, 2015–2017.

**Variables**	**Subcategories**	**Incident functional disability**		***P*-value**
		**No (*n =* 3364)**	**Yes (*n =* 163)**	
		***N* (%)**	***N* (%)**	
Age (in years)	45–54	656 (98.2)	12 (1.8)	<0.001
	55–64	955 (97.8)	21 (2.2)	
	66–74	869 (95.8)	38 (4.2)	
	75 or more	884 (90.6)	92 (9.4)	
Sex	Female	1,790 (95.2)	90 (4.8)	0.616
	Male	1,574 (95.6)	73 (4.4)	
Education	None	196 (92.5)	16 (7.5)	0.003
	Elementary	2,604 (95.1)	133 (4.9)	
	>Elementary	558 (97.7)	13 (2.3)	
Marital status	Not married	1,321 (94.0)	84 (6.0)	0.002
	Married/cohabiting	2,042 (96.3)	79 (3.7)	
Income quartile	Low	782 (93.5)	54 (6.5)	<0.001
	Lower middle	814 (93.9)	53 (6.1)	
	Upper middle	900 (96.4)	34 (3.6)	
	High	868 (97.5)	22 (2.5)	
Alcohol use	Never	2,698 (95.0)	142 (5.0)	0.012
	Past	237 (94.8)	13 (5.2)	
	Current	429 (98.2)	8 (1.8)	
Smoking tobacco use	Never	2,688 (95.4)	129 (4.6)	0.747
	Past	257 (94.5)	15 (5.5)	
	Current	419 (95.7)	19 (4.3)	
Physical activity	None	1,915 (94.8)	105 (5.2)	0.002
	1–149 min/week	871 (94.9)	47 (5.1)	
	≥150 min/week	578 (98.1)	11 (1.9)	
Body mass index	Normal	1,135 (95.3)	56 (4.7)	0.011
	Under	302 (92.1)	26 (7.9)	
	Overweight	623 (96.7)	21 (3.3)	
	Obesity	978 (95.7)	44 (4.3)	
Social engagement	No	225 (96.6)	8 (3.4)	0.371
	Yes	3,137 (95.3)	155 (4.7)	
Multimorbidity (≥2 vs. 0–1)	No	2,605 (96.0)	109 (4.0)	0.002
	Yes	759 (93.4)	54 (6.6)	
Multimorbidity	0	1,599 (96.6)	57 (3.4)	<0.001
	1	1,006 (95.1)	52 (4.9)	
	2	537 (94.5)	31 (5.5)	
	3 or more	222 (90.6)	23 (9.4)	

### Odds ratios for bidirectional associations between functional disability and multimorbidity

In the final logistic regression model adjusted for education, income, age, marital status, sex, smoking tobacco, BMI, alcohol use, physical activity, and social engagement, functional disability at baseline was positively associated with incident MM (≥2) (adjusted OR [aOR]: 2.58, 95% CI: 1.42–4.72), and MM (≥3) at baseline was positively associated with incident functional disability (aOR: 1.97, 95% CI: 1.13–3.43) (refer to Table 4).

**Table 4:** Odds ratios for bidirectional associations between functional disability and multimorbidity (MM).

**Baseline variable**	**Follow-up variable**	**Model 1**		**Model 2**		**Model 3**	
		**OR (95% CI)**	***P*-value**	**OR (95% CI)**	***P*-value**	**OR (95% CI)**	***P*-value**
Functional disability	MM	Odds ratios for the association between functional disability at baseline and incident MM					
Yes	≥2	1 (Reference) 2.32 (1.92–5.44)	<0.001	1 (Reference) 2.47 (1.45–4.22)	<0.001	1 (Reference) 2.58 (1.42–4.72)	0.002
MM	Functional disability	Odds ratios for the association between MM at baseline and incident functional disability					
0 1 2 3 plus	No Yes	1 (Reference) 1.45 (0.99–2.13) 1.62 (1.03–2.54) 2.91 (1.76–4.81)	0.058 0.035 < 0.001	1 (Reference) 1.16 (0.78–1.72) 1.22 (0.77–1.92) 1.95 (1.16–3.26)	0.459 0.404 0.012	0 (Reference) 1.23 (0.81–1.86) 1.15 (0.70–1.89) 1.97 (1.13–3.43)	0.325 0.593 0.017

Model 1: unadjusted; Model 2: adjusted for age, sex, marital status, education, and income; Model 3: adjusted for Model 2 variables plus body mass index, physical activity, smoking, alcohol use, and social engagement.

## Discussion

The first longitudinal study investigates the bidirectional associations between MM and functional disability in Southeast Asia. Consistent with two studies in China and Europe (7), we found that MM (hypertension, diabetes, lung diseases, emphysema, cardiovascular diseases, heart diseases, heart failure, rheumatism, arthritis, bone diseases, low bone density, osteoporosis, kidney diseases, cancer, liver diseases, emotional/nervous or psychiatric diseases, brain diseases, Alzheimer's disease, and visual and hearing impairment) and functional disability were bidirectionally associated with middle-aged and older adults in Thailand. These associations were independent of BMI, sex, marital status, age, education, income, smoking, physical activity, alcohol use, and social engagement.

We found some differences between the present study and the two previous studies (CHARLS and SHARE), namely, the associations between baseline functional disability and incident MM, and the associations between baseline MM and incident functional disability were weaker in this study than in CHARLS and SHARE (7). We believe that the major contributor to this difference was due to the significantly lower sample size in our study compared to CHARLS and SHARE, which includes a shorter follow-up period (2 years) compared to the CHARLS and SHARE study (4 years). In addition, although the type and number of morbidities and covariates assessed in this study were similar to CHARLS and SHARE, covariates in our study may have had a differential effect. For example, after including all covariates in the model, the effect of MM (3+) on functional disability was reduced from 2.9 to 2.0.

In a systematic review, the main consequences of MM were disability and functional decline (48), which may occur due to damage in multiple organs and systems (7, 58). Conversely, older adults with a functional disability may engage in less health behavior, such as physical activity, are less likely to access and adhere to medical care independently, have a higher BMI, and experience more psychological distress than those without functional disability (7). Another possibility is that specific mechanisms of biological aging influence both MM and functional disability. For example, physical inactivity increases both MM and functional disability (49). This leaves the question of possible shared modifiable risk factors for both functional disability and MM, which is subject to further research.

This study found among individuals aged 45 years and older, a prevalence of MM of 23.5%, which is higher than in a study among older adults (≥60 years) in southern Thailand (16.8%) (16) and in a national survey among older adults (≥60 years) in Thailand (14.7%) (17). The lower rate of MM in the latter study may be attributed to fewer morbidities (six) included in the survey (17). The prevalence of MM (23.5%) was higher than in China (17.4%) and Ghana (16.6%), similar to South Africa (23.4%) and Russia (23.6%), lower than in India (25.2%) and Mexico (45.3%) (15), and much lower than among predominantly older adults from six LMICs (45.5%) (14). Reasons for some of these differences are attributable to the different number of morbidities included; for example, in the six-country study, a lower prevalence of MM was found with fewer conditions, and in the same study, a higher prevalence was found with a higher number of MM (e.g., the inclusion of vision and hearing impairment). Moreover, a higher morbidity rate may be related to the symptom-based and physical measurements of morbidities, while our study relied only on self-reported healthcare provider diagnosed morbidities.

Furthermore, this study found among individuals aged 45 years and older, a prevalence of functional disability of 3.2% (4.3% of individuals aged 60 years and older), which is lower than in two national surveys in Thailand in 2014 and 2017 (7.6%) (22, 23). A major reason for the almost double higher prevalence of functional disability in these two latter surveys compared to our study may be attributed to the difference in the number of items of the functional disability measure (our study only used 4 items, while the two surveys reported here used 8 items). Measuring functional disability with more items increases the likelihood of finding more functional limitations. Compared to these prevalence rates of functional disability in Thailand (<10%), much higher rates were found among older adults (≥50 years) in the six LMICs, namely, China (16.2%), India (55.7%), Ghana (44.0%), South Africa (38.6%), Mexico (38.8%), and Russia (43.1%) (15).

Furthermore, we found that consistent with previous research (6, 9, 15, 29, 32, 38, 40), both MM and functional disability increased with age, decreased with higher education, and decreased with higher income. In line with previous studies (32), the prevalence of MM was higher in women than in men, while functional disability did not differ by sex, as found previously (29, 38, 39). The prevalence of MM and functional disability was higher among those who were physically inactive, which is consistent with previous research (32, 34, 40, 43, 44). Consistent with previous studies (32, 35), MM was higher among those with a higher BMI (obesity) and consistent with a study in India (59), underweight was higher among those with functional disabilities. Furthermore, we found that past smoking and past alcohol use were higher among those with MM and functional disability, meaning that individuals with MM and/or functional disability may have stopped smoking and/or alcohol use. Contrary to some previous research (15, 36, 37, 44), we did not find that MM and functional disability decreased social engagement. This result may be related to the overall very high prevalence of social engagement (>93%), and a stricter measure of social engagement could have produced different results.

### Strengths and limitations of the study

The study used a national cohort study with large sample size and adjusted for various confounding social, health, and demographic factors. Study limitations include that MM was assessed by self-reported diagnosed chronic conditions, and functional disability was only measured with a modified shorter version of the ADL scale. We could have distinguished between milder and more severe functional disabilities due to the small sample sizes. Moreover, this study had a 2-year follow-up period, hindering us from measuring long-term associations.

## Conclusion

Baseline MM (≥3) increases the risk of incident functional disability and baseline functional disability increases the risk of incident MM (≥2) among middle-aged and older adults in Thailand. Given these findings, health services should be reoriented to tailor interventions to people with MM to prevent and control future functional disabilities, and interventions targeting people with functional disabilities may help prevent and control MM in middle and late adulthood in Thailand.

## Data availability statement

Publicly available datasets were analyzed in this study. This data can be found here: Data is publicly available at Gateway to Global Aging Data, Health, Aging, and Retirement in Thailand: https://g2aging.org/?section=study&amp;studyid=44.

## Ethics statement

The studies involving human participants were reviewed and approved by Ethics Committee in Human Research, National Institute of Development Administration—ECNIDA (ECNIDA 2020/00012). The patients/participants provided their written informed consent to participate in this study.

## Author contributions

SP, KP, and DA contributed to the design, implementation of the research, and wrote the manuscript. KP analyzed the results. All authors contributed to the article and approved the submitted version.

## Funding

The Health, Aging, and Retirement in Thailand (HART) study is sponsored by the Thailand Science Research and Innovation (TSRI) and the National Research Council of Thailand (NRCT).

## Conflict of interest

The authors declare that the research was conducted in the absence of any commercial or financial relationships that could be construed as a potential conflict of interest.

## Publisher's note

All claims expressed in this article are solely those of the authors and do not necessarily represent those of their affiliated organizations, or those of the publisher, the editors and the reviewers. Any product that may be evaluated in this article, or claim that may be made by its manufacturer, is not guaranteed or endorsed by the publisher.
